# Milk Quality and Safety in a One Health Perspective: Results of a Prevalence Study on Dairy Herds in Lombardy (Italy)

**DOI:** 10.3390/life12060786

**Published:** 2022-05-25

**Authors:** Valerio M. Sora, Sara Panseri, Maria Nobile, Federica Di Cesare, Gabriele Meroni, Luca M. Chiesa, Alfonso Zecconi

**Affiliations:** 1 Department of Biomedical, Surgical and Dental Sciences, One Health Unit, School of Medicine, University of Milan, Via Pascal 36, 20133 Milan, Italy; valerio.sora@unimi.it (V.M.S.); gabriele.meroni@unimi.it (G.M.); 2 Department of Clinical and Community Sciences, School of Medicine, University of Milan, Via Celoria 22, 20133 Milan, Italy; 3 Department of Veterinary Medicine and Animal Sciences, University of Milan, Via Università 6, 26900 Lodi, Italy; sara.panseri@unimi.it (S.P.); maria.nobile@unimi.it (M.N.); federica.dicesare@unimi.it (F.D.C.); luca.chiesa@unimi.it (L.M.C.)

**Keywords:** milk quality, dairy herds, one health approach, antimicrobial residues

## Abstract

Mastitis is one of the major diseases of dairy cows that affects milk quality and quantity and increases the potential risk for the presence of antimicrobial residues (AR) in milk, which could lead to the development of antimicrobial resistance (AMR) among human pathogens. Even if the presence of AR in milk and milk products is low in many countries, the threat is not negligible and cannot be ignored. These problems may be investigated by applying a One Health approach, and this prevalence study aimed to estimate the risks for human health related to milk production applied to dairy herds in Lombardy. Three hundred thirty-one bulk tank milk samples were randomly collected and analyzed by CombiFoss 7 and MilkoScan 7 (milk quality, bacteria, and somatic cell count), an HPLC system coupled to a Q-Exactive Orbitrap (AR), and qPCR (contagious pathogens). The data were analyzed by a generalized linear model. The results showed a relatively high prevalence of contagious pathogens (*S. aureus* 28.1%; *Str. agalactiae* 7.3%; *M. bovis* 3%), which primarily affect milk nutritional components decreasing mainly milk fat content (range 1%–2.5%), but did not show them to be associated to an increase of the risk of antimicrobial residues. These latter ones were recovered only in 7/331 samples at concentrations far below official MLRs. The results support currently active surveillance programs’ efficacy in reducing AR risks, which may be further improved by prioritizing them based on geographical area characteristics.

## 1. Introduction

Milk production represents a significant food source for human consumption and an important part of the gross national product in many countries, including Italy. Moreover, many people are directly or indirectly occupied in the milk production chain. Besides the positive economic and social values, the milk production chain may also represent a potential risk to human health. Indeed, as described by ref. [1] in a One Health perspective, milk production should be based on the health and welfare of dairy cows [2], on the proper use of antimicrobials [3], and on food safety that includes both the absence of zoonotic pathogens and contaminants [4]. When a One Health approach is applied to milk production assessment, the antimicrobial resistance problem is one of the most important to be considered, and it is strictly connected with mastitis, which is a major disease both for its prevalence and for its costs [5,6,7], and the leading cause of treatment with antimicrobials in dairy cattle [8,9]. On the other side, the availability of safe milk and milk products improves the quantity and quality of nutrients for human consumption and promotes human health [10,11].

Antimicrobial residues (AR) are a primary concern worldwide, being related to the development of antimicrobial resistance (AMR) among human pathogens [12,13,14]. Though the presence of AR in milk and milk products is low in many countries, including Italy, the threat is not negligible and cannot be ignored [15].

Among mastitis pathogens, contagious ones (*Streptococcus agalactiae*, *Staphylococcus aureus,* and *Mycoplasma bovis*) are considered within the major threats for milk safety and quality [16,17]. Moreover, *Str. agalactiae* has been considered for a long time as a zoonotic agent [18,19], even if a recent study on their genetics suggests an opposite relationship with the spread of human strains in the dairy cattle population [20]. *S. aureus* is also considered a zoonotic agent, mainly when Methicillin-resistant S. *aureus* (MRSA) strains are considered [21], even if the frequency of MRSA in milk has a large variability among studies [21,22,23,24].

Health problems and, principally, mastitis also affect milk quality and cheese yield due to decreased casein concentration and a lower attitude to ripening [25,26].

About 45% of Italian milk is produced in Lombardy [27], a region located in the northern part of Italy. This large milk production and the related high number of cows emphasized the importance of accurate monitoring of milk quality and safety. This monitoring is even more important from a One Health approach because Lombardy is high-density populated (418/Km^2^), and the human-animal interface is quite wide.

The presence of AR in bulk milk is checked with high frequency by dairies following current EU regulations. This guarantees consumers that the milk and milk products do not contain an amount of AR potentially dangerous for human health. However, this does not mean that AR are absent from the products, and this may represent a potential risk of the development of AMR in humans. These residues may be identified nowadays even when their concentrations are much lower than the official maximum residue limits (MRL) [28].

The study aimed to estimate the presence of hazards to human health related to milk production through a prevalence study on dairy herds in Lombardy by applying a One Health approach. More specifically, we investigated the presence of hazards as contagious bacteria in bulk tank milk (BTM), the relationship of their presence with milk quality, and the presence of AR in BTM with molecular and chromatographic methods.

## 2. Materials and Methods

### 2.1. Sampling Design

The study was designed as a prevalence study and the sample size was calculated assuming a prevalence of contagious pathogens of 30% on a total dairy herd population of 4931 herds [29]. The total amount of samples was distributed proportionally based on the number of dairy herds in each of the 12 Lombardy provinces (see Supplementary Figure S1).

### 2.2. Milk Sample Collection

Three hundred thirty-one raw bovine milk samples were randomly collected among the 931 dairy herds, each divided into two bottles of 500 mL, one delivered refrigerated for molecular and quality assays and the other one frozen and stored at −20 °C until being analyzed for antimicrobial residues.

### 2.3. Milk Quality Assay

After collection, milk samples were placed at 4 °C immediately and analyzed within 24 h. Milk fat and protein were measured using MilkoScan 7 (calibrated according to ISO 9622/IDF 141:2013), while somatic cell counts (SCC) were determined by Fossomatic 7DC (according to ISO 13366-2/IDF 148-2:2006 standards). The SCC was Log_10_ transformed for further analysis.

### 2.4. Contagious Pathogen Assay

Milk samples were analyzed using qPCR through a commercial diagnostic kit (Mastitis 4E kit; DNA Diagnostic A/S) according to the manufacturer’s instructions. This technique showed sensitivity and specificity, respectively, of ≥0.95 and ≥0.99 for the contagious pathogens [30,31,32]. This kit allows bacterial DNA extraction, identification, and quantification of *S. aureus*, *Str. agalactiae M. Bovis*, and *Prototheca* spp. This latter pathogen, being of environmental origin, was not considered further in this paper. The reaction conditions of qPCR were as follows: 95 °C for 1 min, 40 amplification cycles at 95 °C for 5 s and 60 °C for 25 s. The cycle threshold (Ct) values were considered positive when the values were ≤37, as suggested by the manufacturer. The qPCR reactions were performed on Stratagene Mx3005P (Agilent Technologies Inc., Santa Clara, CA, USA).

### 2.5. Antimicrobial Residues Assay

#### 2.5.1. Antibiotic Extraction for HPLC-HRMS Analysis

The sample extraction for the antibiotic confirmatory method was performed following the analytical protocol of Chiesa et al. [28,33]. Briefly, an aliquot of 1 mL of raw bovine milk was spiked at 2 ng mL^−1^ with the internal standard (IS), added of 100 μL, 20% *w*/*v* of Tri-chloroacetic acid for protein precipitation, and extracted with 5 mL of McIlvaine buffer (pH 4.0). After sonication for 10 min and centrifugation (2500× *g*, 4 °C, 10 min), the sample was defatted with hexane (2 × 3mL), and then purified by Oasis HLB SPE. The SPE was pre-conditioned with 3 mL of methanol and 3 mL of Milli-Q water; the extract was loaded and then washed with 2 × 3 mL methanol:water (5:95 *v*/*v*) before the final elution with 5 mL of methanol. The eluate was dried and reconstituted in 200 μL of methanol: 0.1%formic acid (10:90 *v*/*v*).

#### 2.5.2. HPLC-HRMS Parameters for Confirmatory Analysis

Instrumental analyses were performed by an HPLC system coupled to a Q-Exactive Orbitrap (Thermo Fisher Scientific, San Jose, CA, USA). A Synergi Hydro-RP reverse-phase column (150 × 2.0 mm, i.d. 4 µm), with a C18 guard column (4 × 3.0 mm; Phenomenex, Torrance, CA, USA) was used and the mobile phase consisted of a binary mixture of Phase A (aqueous HCOOH 0.1%) and B (MeOH). The chromatographic gradient and the mass parameter set for the full scan acquisition combined with an independent data acquisition for the confirmatory response were well described in the previous work of Chiesa et al. [34].

#### 2.5.3. HPLC-HRMS Method Validation

The HPLC-HRMS method was fully validated according to Commission Decision 2002/657/EC guidelines and SANCO/2004/2726 revision 4, as reported in Chiesa et al. [33]. The recovery, the decision limit (CCα) and detection capability (CCβ), and precision, in terms of intra- and inter-day repeatability, were assessed in compliance with the recommended tolerance ranges of European guidelines.

#### 2.5.4. Chemicals and Reagents

All solvents of analytical LC-MS grade were provided by Merck (Darmstadt, Germa-ny). Sixty-six antibiotics belonging to different classes: Enrofloxacin, Difloxacin, Danofloxacin, Levofloxacin, Lomefloxacin, Marbofloxacin, Norfloxacin, Enoxacin, Flumequine, Nadifloxacin, Oxolinic acid, Nalidixicacid, Amoxicillin, Ampicillin, Phe-noxymethylpenicillin, Benzylpenicillin, Cefadroxil, Cefalexin, Cefalonium, Cefalothin, Cefazolin, Cefoperazone, Cefquinome, Cefapirin, Ceftiofur, Desfuroylceftiofur, Cloxacillin, Dicloxacillin, Benethamine penicillin, Nafcillin, Oxacillin, Piperacillin, Tylosin, Tilmicosin, Oleandomycin, Spiramycin, Neospiramycin, Kitasamycin, Josamycin, Tu-lathromycin, Erythromicyn A, Rifaximin, Sulfadiazine, Sulfadimethoxine, Sulfadimidine, Sulfamerazine, Sulfamethoxazole, Sulfamonomethoxine, Sulfapirydine, Sulfatiazole, Tri-methoprim, Chlorotetracycline, Oxytetracycline, Tetracycline, Doxyicline, Lincomycin, Chloramphenicol, Tiamphenicol, Florfenicol, Florfenicol amine, Tiamulin, Valnemulin, Dimetridazole, Ronidazole, Tinidazole), and Enrofloxacin-d5 (internal standard) were bought from Merck (Darmstadt, Germany).

The Solid Phase extraction cartridges SPE Oasis HLB (3 mL, 60 mg) were provided by Waters (Milford, MA, USA).

### 2.6. Statistical Analysis

Data descriptions were calculated by XLSTAT 2022 1.2 (Addinsoft, New York, NY, USA). Data were also analyzed by ANOVA using a generalized linear model applying the GLM procedure of SAS 9.4 (Sas Institute, Cary, NC, USA). The model was:

Yijkwz = µ + Di + Vz + Aj + Sk + Mw + (Aj + Sk + Mw) × Di + (Aj + Sk + Mw) × Vz + eijkwz

where Y = dependent variables (TBC, SCC, Fat%, Protein%); µ = general mean; Di = effect of the the district (i = 1–3); Vz = effect of herd size (z = 1–5); (Aj = effect of *Str. agalactiae* results (j = negative, positive); Sj = effect of *S. aureus* results (j = negative, positive); Mw = effect of *M. bovis* results (j = negative, positive); (Aj + Sk + Mw) × Di = effect of contagious pathogens nested in the district; (Aj + Sk + Mw) × Vz = effect of contagious pathogens nested in t in herd size, eijkwz = residual error. 

## 3. Results

### 3.1. Sample Description

Overall, 331 bulk milk (BTM) samples from an equal number of dairy herds were collected in 12 provinces of Lombardy and classified into the three major geographical areas (alpine, semi-alpine, and Po valley). The alpine area grouped the provinces of Como, Lecco, and Sondrio; the Po valley area included the provinces of Cremona, Lodi, Milano, Mantova, Monza, and Pavia; the semi-alpine area included the provinces of Bergamo, Brescia, and Varese.

The number of herds for each area, and herd size, are reported in Table 1, while Table 2 reports yearly production and average milk yield. Table 3 reports the data concerning the average fat and protein content of BTM from the sampled herds.

The herd size showed large variability (6–1150 cows/herd) with an average of 191 cows/herd, close to the average of the two areas with the largest cow population (Semi-alpine and Po Valley), as expected.

The yearly milk production also showed large variability, with the lowest amount in the alpine area, where the frequency of small herds is higher, and the highest in herds located in the Po Valley where there is the largest frequency of large herds (Table 2). Individual milk yield showed the same pattern with lower mean production in cows from the alpine area and the highest in cows from Po Valley herds. Milk fat and protein proportions showed the opposite trend with the highest proportions for both parameters in Alpine herds and the lowest in cows from Po Valley herds, values very likely due to the different milk yield per cow, and the prevalent breeds (Brown Swiss in alpine area and Italian Holstein in Po valley herds).

### 3.2. Health Status

The average SCC and total bacteria count (TBC) are reported in Table 4, whereas Table 5 reports the prevalence of the contagious bacteria within the three areas considered.

The SCC values showed an average of 5.34 ± 0.22 Log_10_ cells/mL as the results of the mean for the two area with the largest number of herds, while in the Alpine area the SCC mean values were lower. The values observed were largely below the current threshold defined by the EU (400,000 cells/mL or 5.60 Log_10_ cells/mL).

The TBC values were also relatively close in the range 4.01–4.21 CFU/mL with the lowest values in the Alpine area and the highest in the Semi-alpine one. Additionally, in this case, the values are quite low compared with the EU thresholds (100,000 CFU/mL or Log_10_ 5.00 cells/mL).

When contagious pathogens prevalence was considered, the overall prevalence of *S. aureus* was 28.1% (93 herds), while *Str. agalactiae* was recovered from 7.2% (24 herds), and *M. bovis* only in 3.0% of the herds (10 herds). The frequencies of the three pathogens were different at χ^2^ test (*p* < 0.001) The highest prevalence for *S. aureus* was in the Alpine area, whereas *Str. agalactiae* and *M. bovis* prevalences were higher in the Semi-alpine area. The risk prevalence associated with the production area was assessed using the Po Valley prevalence as reference, and the result showed that the only significant risk ratio was related to *S. aureus* in the Alpine area (risk ratio 1.6 and confidence limits 1.0–1.8). In the other cases the risk ratio was >1, but not significant at χ^2^ test or Fisher exact test (α = 0.05).

### 3.3. Antimicrobial Residues

All 331 raw bovine milk samples were analyzed by the HPLC-HRMS method. Table 6 reports only the samples in which the presence of antibiotic residue was detected. Only one compound was found for each sample listed in the table, and all the molecules recovered from the seven samples had a concentration far below the official maximum residue limits defined by the EU [35]. As an example, a chromatogram and its high-resolution mass spectrum of a sample in which lincomycin was found, compared with a negative sample, is shown in Supplementary Figure S2. All the molecules, except tulathromycin, may be used for lactating cows, whereas tulathromycin is not registered for administration to lactating cows, suggesting an unapproved use of a product containing that molecule. The prevalence ratio between the positive samples in herds with contagious pathogens (3/127) and the positive samples in contagious-negative herds (4/204) was numerically >1, but not significant at the Fisher exact test (α = 0.05).

### 3.4. Pathogen-Related Risks for Milk Quality and AMR

The effects due to the presence of contagious pathogens were estimated by a general linear model as described in the material and methods. Herd TBC was uninfluenced by the presence of the pathogens and their interaction with area and herd size. SCC and protein (%) were significantly influenced by *Str. agalactiae* and its interaction with herd size, respectively. Fat (%) showed to be the parameter most influenced by the factors considered (Table 7) and, among pathogens, both *Str. agalactiae* and *M. bovis* showed to influence fat (%) variance. Table 8 reports the mean values for the different levels of the statistically significant factors.

The risk for the presence of AR related to the health status (presence or absence of contagious pathogens), assessed by calculating the prevalence ratio between the positive samples in herds with contagious pathogens (3/127) and the positive samples in contagious-negative herds (4/204), was numerically >1 (1.2, 0.273–5.275), but not significant at the Fisher exact test (α = 0.05).

## 4. Discussion

The One Health approach is generally associated with severe disease control/prevention or major zoonosis such as tuberculosis and antimicrobial resistance [35,36]. However, this approach may also be useful when applied to investigate the health aspects related to primary food production [1,37]. Indeed, this approach requires that the assessment of the health and actions applied in a specific area (in our case the dairy production) takes in account not only the direct consequences of a potential hazard, but how this hazard (i.e., contagious pathogens) and the control measures would affect human and environmental health (i.e., potential increase in AMR). In simple words, the One Health approach requires a holistic vision of the problem, and, consequently, assessment and actions that include this holistic vision.

Therefore, the improvement of food safety in cow milk production, through the improvement of animal health, and the resulting enhancement of human health may represent a good example of the One Health approach. Milk should be produced in herds that guarantee both the health and welfare of the animals, but milk could be a passive vector of zoonotic bacteria, AMR bacteria, and AR, particularly when cow health does not have a proper health level [3]. Besides, in this latter case, milk quality is lower, and the quantity and quality of nutritional factors is decreased. The importance of any single problem may differ in the different production areas, i.e., zoonotic and nutritional aspects could be more important in areas where cow farming is still suboptimal, whereas AMR and AR may be more frequent in areas with an intensive herd management, but none of the above are completely absent in any farming scenario.

To assess the presence of some of these problems and the related potential risks, a prevalence study was designed and applied to dairy farms in Lombardy, where >40% of Italian milk is produced. The study was focused on milk quality and AR, since the region is free from the major zoonotic bacteria (*Mycobacterium bovis* and *Brucella abortus*).

Lombardy has different orographic areas (Alpine, semi-alpine and Po-valley), which influence both milk quality and quantity [38]. Indeed, the study confirmed that the largest herds are in Po-valley and have the highest milk production both at the herd and cow level. On the other side, Alpine herds, on average, are the smallest and with the lowest production. However, when milk quality is considered, Alpine herds showed to have the highest proportion of fat and protein and the lowest values for SCC and TBC. Therefore, the surveillance’s prioritization should also consider these aspects [39].

### 4.1. Human-Animal Interface

The presence of a relatively high frequency of herds with contagious pathogens (of *S. aureus*, *Str. agalactiae*, *M. bovis*) suggests a potential risk for human health. This aspect may be negligible if the results of recent studies [20,21,22,23,24] are also be confirmed for bacteria isolated in Lombardy. However, the potential transmission of *Str. agalactiae* from humans to cows should also be considered [20]. Moreover, the negative effects of contagious pathogens on the economic sustainability of the affected herds are still present and should be considered [40].

For what concerns the risks for a decrease in milk hygiene (SCC and TBC) and for a decrease in milk quality (fat and protein proportion), the results showed some unexpected results. Indeed, *S. aureus*, despite being the most prevalent pathogen, did not significantly affect milk quality and hygiene. The relatively low prevalence of these bacteria within the positive herds and by the higher frequency of younger cows in Lombardy herds may explain these results [41]. Indeed, the animals could be infected for a shorter time, thus minimizing the effects on milk secretion [42,43,44]. However, the presence of *Str. agalactiae* and *M. bovis* alone or stratified by herd size or orographic area showed to significantly decrease the proportion of milk components in most of the cases and an increased SCC in the case of *Str. agalactiae*. These negative changes support the importance of an appropriate control for these infections to avoid a decrease in the nutritional value of the milk.

### 4.2. Human-Animal-Environment Interface

The assessment of the risk for the presence of AR residues showed only 7 positive samples out of 331 with a concentration largely below the official MRL defined by EU relations. Other studies showed an association between SCC and AR in milk [45], and the absence of this relationship in this study may be explained by the strict surveillance of AR operated by dairies and regional veterinarians. Overall, the risk for human health and environmental contamination seems to be very low or negligible. However, the evidence of an extra-label treatment, even if in a single case, supports the importance of continuous and rigorous surveillance to increase food safety.

## 5. Conclusions

This study attempted to apply in practice the One Health approach to milk production by taking in consideration both the presence of hazards and their consequences on human and potentially environmental health. The prevalence study conducted in Lombardy dairy herds showed that the One Health approach is suitable for this application. The results showed a relatively high prevalence of contagious pathogens, which primarily affect milk nutritional components and herd sustainability, but do not increase the risk of antimicrobial residues. The positive results support currently active surveillance programs’ efficacy, which may be further improved by prioritizing them based on geographical area characteristics.

## Figures and Tables

**Table 1:** Distribution of bulk tank milk samples among the three major geographical areas in Lombardy and the related herd size.

Area	N	%	Number of Lactating Cows (N)		
			Mean	Min	Max
Alpine	28	8.5	87.7	9	251
Semi-alpine	153	46.2	188.6	6	1150
Po valley	150	45.3	213.0	23	947
**TOTAL**	**331**	**100.0**	**191.1**	**6**	**1150**

**Table 2:** Total yearly production and average cow milk yield/year in the sampled herds classified by the three major geographical areas in Lombardy.

Area	Milk Production (tons/year)			Average Milk Production (kg/year)		
	Mean	Min	Max	Mean	Min	Max
Alpine	817.6	25.7	2392.2	7826.6	1284.8	11,763.7
Semi-alpine	1869.8	73.5	8362.9	10,982.0	4777.7	12,553.7
Po valley	2507.5	188.7	9259.0	11,112.7	3686.0	29,314.0
**TOTAL**	**2059.2**	**25.7**	**9259.0**	**10,783.0**	**1284.8**	**29,314.0**

**Table 3:** Average proportion of fat and protein in bulk tank milk from the sampled herds classified by the three major geographical areas in Lombardy.

Area	Fat (%)		Protein (%)	
	Mean	Std.dev.	Mean	Std.dev.
Alpine	4.12	0.18	3.57	0.16
Semi-alpine	4.01	0.17	3.47	0.10
Po valley	3.99	0.17	3.49	0.09
**TOTAL**	**4.01**	**0.18**	**3.48**	**0.11**

**Table 4:** Somatic cells and total bacteria counts (means ± standard deviation) in bulk tank milk sampled from dairy herds classified by the three major geographical areas in Lombardy.

Area	SCC ^1^ (Log_10_ SCC × 1000/mL)		TBC ^2^ (Log_10_ TBC × 1000/mL)	
	Mean	Std. Deviation	Mean	Std. Deviation
Alpine	5.23	0.20	4.01	0.38
Semi-alpine	5.34	0.27	4.25	0.44
Po valley	5.34	0.13	4.21	0.29
**TOTAL**	**5.34**	**0.22**	**4.21**	**0.38**

^1^ SCC: somatic cell count. ^2^ TBC: total bacteria count.

**Table 5:** Estimated prevalence of contagious pathogens in bulk tank milk sampled from dairy herds classified by the three major geographical areas in Lombardy.

Area	*S. aureus*		*Str. agalactiae*		*M. bovis*	
	%	Conf.lim.95%	%	Conf.lim.95%	%	Conf.lim.95%
Alpine	39.3	21.2–57.4	7.1	0–16.7	0	0–0
Semi-alpine	30.1	22.8–37.3	8.5	4.1–12.9	3.9	0.8–7.0
Po valley	24.0	17.1–30.1	6.0	2.2–9.8	2.7	0.1–5.2
**TOTAL**	**28.1**	**23.3–32.9**	**7.3**	**4.6–10.0**	**3.0**	**0.1–5.2**

**Table 6:** Samples in which antibiotic residues were detected and relative concentration (ng/mL).

Animal Codex	Concentration (ng/mL)					
	Tulathromycin	Lincomycin	Enrofloxacin	Nalidixic Acid	Sulfadimidine	Oxytetracycline
870104	18.11 ± 0.03	n.d ^1^	n.d	n.d	n.d	n.d
08/71117	n.d	115.88 ± 0.05	n.d	n.d	n.d	n.d
12/29167	n.d	18.92 ± 0.04	n.d	n.d	n.d	n.d
0971094	n.d	n.d	<CCβ ^2^	n.d	n.d	n.d
10/23702	n.d	n.d	n.d	<CCβ	n.d	n.d
11/64650	n.d	n.d	n.d	n.d	2.43 ± 0.02	n.d
0972102	n.d	n.d	n.d	n.d	n.d	12.93 ± 0.04

^1^ n.d. = not detected. ^2^ detection capability CCβ = tulathromycin (0.51 ng/mL), lincomycin (0.20 ng/mL), enrofloxacin (0.23 ng/mL), nalidixic acid (0.24 ng/mL), sulfadimidine (0.25 ng/mL), oxytetracycline (0.22 ng/mL).

**Table 7:** Results of analysis of variance of the GLM model estimating the effects of the presence of contagious pathogens and their interactions with herd sizes and areas on milk quality parameters. The table reports only the factors significantly affecting fat, protein, and SCC variances.

Fat (%)	Protein (%)	SCC (Log_10_ SCC * 1000/mL)
Area *	Herd size x *Str. Agalactiae* **	*Str. Agalactiae* *
*Str. Agalactiae* *		
*M. bovis*		
Herd size × *Str. Agalactiae* * Area × *M. bovis* **		

* *p* < 0.10 ** *p* < 0.05.

**Table 8:** Mean values for milk components and SCC for the factors and their interaction which resulted as significant at variance analysis of the GLM.

Factors	Level	Fat (%)	Protein (%)	SCC (Log10 SCC × 1000/mL)
*Area*	Alpine	4.11 ^a,^*		
	Semi-alpine	4.01 ^a,b^		
	Po valley	3.99 ^b^		
*Str. agalactiae*	Positive	3.98 ^a^		5.39 ^a^
	Negative	4.01 ^b^		5.33 ^b^
*M. bovis*	Positive	3.96 ^a^		
	Negative	4.07 ^b^		
*Str. agalactiae* × herd size				
6–60 cows	Positive	4.09 ^a^	3.56 ^a^	
	Negative	4.12 ^a^	3.51 ^a^	
61–120 cows	Positive	3.62 ^a^	3.36 ^a^	
	Negative	4.02 ^b^	3.51 ^b^	
121–180 cows	Positive	4.02 ^a^	3.40 ^a^	
	Negative	4.01 ^a^	3.48 ^a^	
181–240 cows	Positive	4.15 ^a^	3.53 ^a^	
	Negative	4.02 ^a^	3.47 ^a^	
>240 cows	Positive	3.96 ^a^	3.42 ^a^	
	Negative	3.95 ^a^	3.47 ^a^	
*M. bovis* × area				
Semi-alpine	Positive	4.07 ^a^		
	Negative	4.00 ^b^		
Po-valley	Positive	3.79 ^a^		
	Negative	3.99 ^b^		

* cells with different superscript (a,b) within each factor level (positive/negative) are statistically different at α = 0.05.

## Supplementary Materials

The following supporting information can be downloaded at: https://www.mdpi.com/article/10.3390/life12060786/s1.
