# Supplementary material for: Milk Quality and Safety in a One Health Perspective: Results of a Prevalence Study on Dairy Herds in Lombardy (Italy)
Source: Life (Basel). 2022 May 25;12(6):786. doi: 10.3390/life12060786 (PMC9225654; doi:10.3390/life12060786)
Supplement: Supplementary file 1 [file life-12-00786-s001.zip › life-1725766-supplementary.pdf]

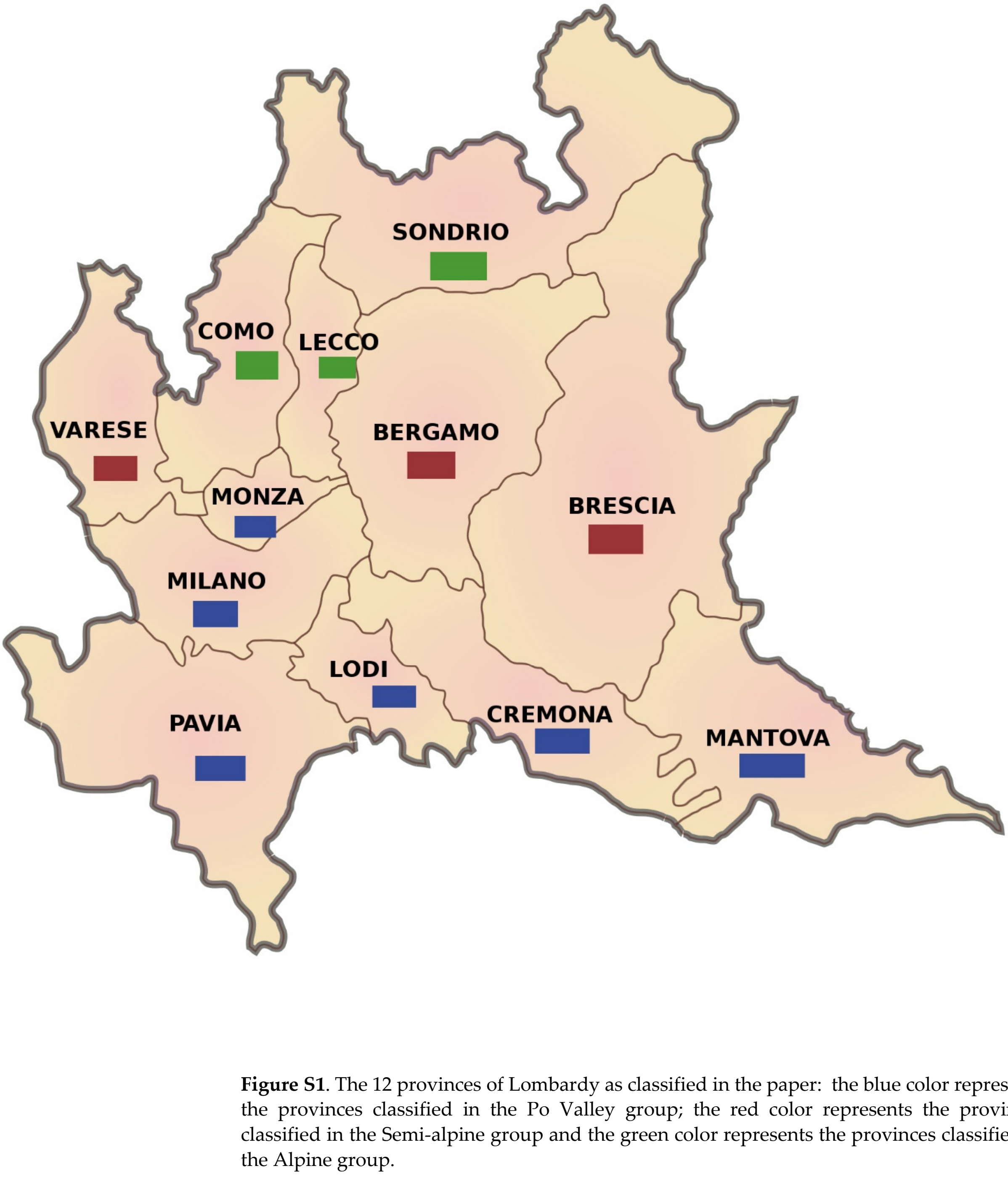

Figure S1. The 12 provinces of Lombardy as classified in the paper: the blue color represents the provinces classified in the Po Valley group; the red color represents the provinces classified in the Semi-alpine group and the green color represents the provinces classified in the Alpine group.

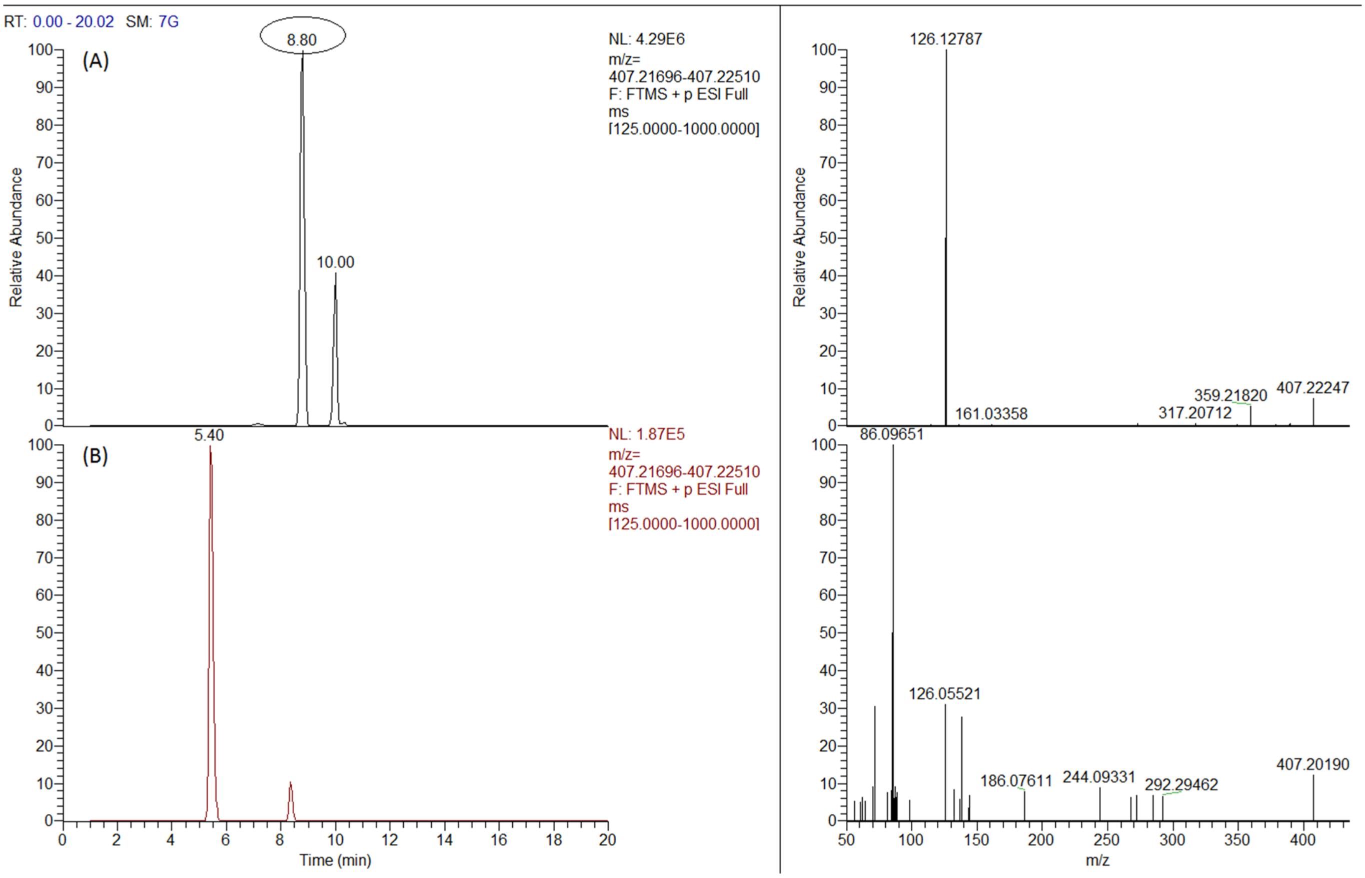

Figure S2. Extracted chromatogram and mass spectrum of lincomycin found in one raw bovine milk sample (A) compared with a negative sample (B).
